# Mini-Review on Bioinspired Superwetting Microlens Array and Compound Eye

**DOI:** 10.3389/fchem.2020.575786

**Published:** 2020-09-29

**Authors:** Jiale Yong, Hao Bian, Qing Yang, Xun Hou, Feng Chen

**Affiliations:** ^1^State Key Laboratory for Manufacturing System Engineering and Shaanxi Key Laboratory of Photonics Technology for Information, School of Electronic Science and Engineering, Xi'an Jiaotong University, Xi'an, China; ^2^School of Mechanical Engineering, Xi'an Jiaotong University, Xi'an, China

**Keywords:** microlens array, artificial compound eye, superhydrophobicity, underwater superoleophobicity, liquid repellence, self-cleaning, anti-fogging

## Abstract

Microlens arrays (MLAs) and MLA-based artificial compound eyes (ACEs) are the important miniaturized optical components in modern micro-optical systems. However, their optical performance will seriously decline once they are wetted by water droplets (such as fog, dew, and rain droplets) or are polluted by contaminations in a humid environment. In this mini-review, we summarize the research works related to the fabrication of superwetting MLAs and ACEs and show how to integrate superhydrophobic and superoleophobic microstructures with an MLA. The fabrication strategy can be split into two categories. One is the hybrid pattern composed of the MLA domain and the superwetting domain. Another is the direct formation of superwetting nanostructures on the surface of the microlenses. The superhydrophobicity or superoleophobicity endows the MLAs and ACEs with liquid repellence and self-cleaning function besides excellent optical performance. We believe that the superwetting MLAs and ACEs will have significant applications in various optical systems that are often used in the humid or liquid environment.

## Introduction

Microlens arrays (MLAs) and MLA-based artificial compound eyes (ACEs) play a key role in advanced micro-optical systems (Pan et al., 2007; Song et al., 2013; Gorzelak et al., 2014; Petsch et al., 2016; Lin et al., 2018). By taking advantages of small size, high integration, and striking optical capability, MLAs and ACEs are widely applied in light-field regulation (Deng et al., 2014; Wei et al., 2018; Zhou et al., 2018; Sohna et al., 2019), fiber coupling (Elsherif et al., 2019; Liu et al., 2019; Orth et al., 2019), lab-on-chip devices (Fei et al., 2011; Lv et al., 2016; Vespini et al., 2016), biochemical observation (Ma et al., 2014; Holzner et al., 2018), laser microfabrication (Bekesi et al., 2010; Li et al., 2020), solar cells (Chen Y. et al., 2013), sensors (Zanella et al., 2020), three-dimensional imaging (Li et al., 2016; Kim et al., 2019; Zhang et al., 2019; Joo et al., 2020; Qin et al., 2020; Zhao et al., 2020), and light extraction (Shin et al., 2018; Zhou et al., 2019a,b). However, the normal use of the MLA is usually restricted by many limitations. For example, when the traditional MLA works outdoors, dust will soon deposit on the MLA surface. For a humid environment, liquid droplets (such as fog, dew, and rain) can easily pin on the MLA surface. Once the MLA is wetted or polluted, its optical performance will seriously decline. To maintain good imaging ability, the regular maintenance and clean for an MLA is highly required. Frequent clean using the lens wiping paper may result in damage to the fine optical microstructure on the MLA surface, thus shorting the lifetime of MLA. The use of detergents and organic solvents to remove dust and contaminations will cause environmental pollution. Sometimes, the MLA-based components need to be disassembled from the optical system to remove the adhered water droplets or contaminations. The abovementioned problems caused by the adhered droplets and contaminations can be avoided if the MLAs have a great liquid-repellent ability and self-cleaning function. Fabrication of such superwetting MLAs has important implications from a practical point of view, but there are still no articles that summarize the methods of combining superwetting microstructures with MLAs.

Here, the strategy of fabricating superwetting MLAs and ACEs is discussed and summarized. This mini-review aims to show how to integrate superhydrophobic and superoleophobic microstructures with an MLA. The article starts with a brief introduction of the significance of endowing an MLA with liquid repellence and self-cleaning ability. Then, some examples are given to present the typical superwettability in nature, such as superhydrophobicity and underwater superoleophobicity. The next part shows the recent research works related to the fabrication of superwetting MLAs and ACEs. The fabrication strategy is split into two categories. Finally, a brief discussion of current challenges and prospects in the fabrication and applications of the superwetting MLAs and ACEs is provided in our perspective.

## Superwettability

**Figure 1:**
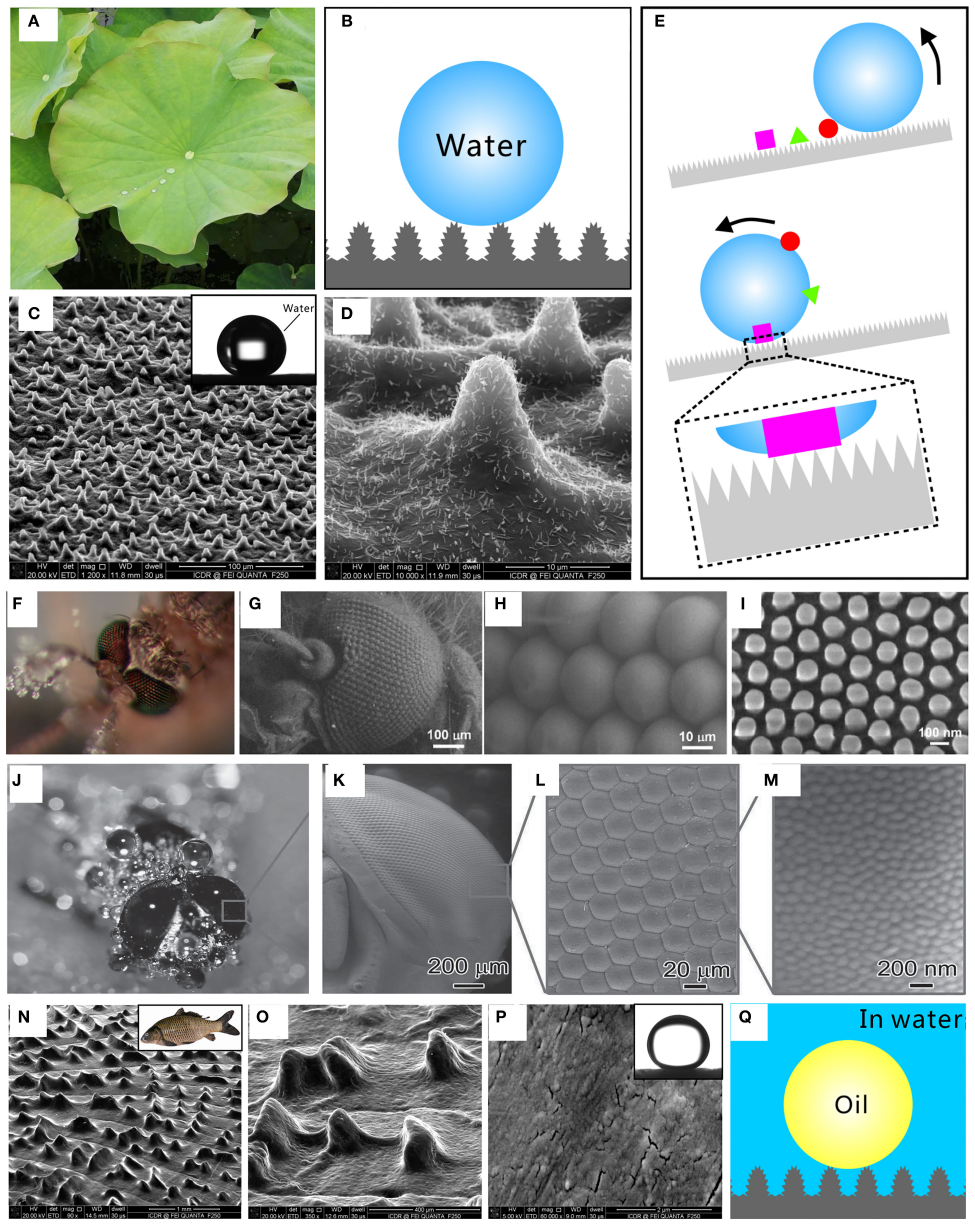
Superwettability in nature. Superhydrophobicity and self-cleaning effect of a lotus leaf surface: **(A)** photo of lotus leaves, **(B)** Cassie wetting state between the water droplet and the microstructure of a lotus leaf, **(C,D)** SEM images of the microstructures on the lotus leaf surface, and **(E)** schematic of the self-cleaning effect of a superhydrophobic surface. The inset in **(C)** shows a water droplet on the surface of a lotus leaf. Anti-fogging property of mosquito's eyes: **(F)** photo of a mosquito blown with fog, **(G)** SEM image of the mosquito compound eye, **(H)** SEM image of ommatidia on a mosquito eye, and **(I)** SEM image of the fine nanoscale nipples on the surface of mosquito ommatidia. Anti-fogging property of fly eyes: **(J)** photo of a fly blown with fog, **(K,L)** SEM images of a fly compound eye, and **(M)** SEM image of the nanoscale protuberances on the ommatidia of the fly compound eye. Underwater superoleophobicity of fish scales: **(N,O)** SEM images of the fish scale surface covered with hill-like microstructures, **(P)** SEM image of the nanoscale bleb structures on the hill-like microstructure of fish scale, **(Q)** underwater Cassie wetting state between an oil droplet and the surface microstructures of fish scale in a water medium. The inset in **(N)** is the photo of a fish, and the inset in **(P)** shows an underwater oil droplet on a fish scale. Reproduced from Yong et al. (2017a) with the permission of the American Chemical Society. Reproduced from Yong et al. (2014b) with the permission of the Royal Society of Chemistry. Reproduced from Gao et al. (2007) and Sun et al. (2014) with the permission of the WILEY-VCH. Reproduced from Yong et al. (2018a) with the permission of Yong et al.

Many animals and plants have developed colorful superwetting surface microstructures through evolution (Barthlott and Neinhuis, 1997; Parker and Lawrence, 2001; Gao and Jiang, 2004; Zheng et al., 2007, 2010; Liu et al., 2009; Ju et al., 2012; Chen F. et al., 2013; Yong et al., 2017a, 2018a). Lotus leaf has a great ability to repel water droplets and self-clean its surface because of the excellent superhydrophobicity (Figure 1A; Barthlott and Neinhuis, 1997; Yong et al., 2017a). Raindrops or dewdrops have a sphere shape on the lotus leaf with the water contact angle (CA) above 150° (inset of Figure 1C) and can easily roll away. Such superhydrophobicity is attributed to the combination of the hierarchical surface microstructures and the low-surface-energy chemical composition of the lotus leaf (Bellanger et al., 2014; Jiang et al., 2015; Wen et al., 2015; Su et al., 2016; Yong et al., 2017b,c, 2018b; Bai et al., 2020). The scanning electronic microscopy (SEM) image reveals that plenty of papilla structures with a diameter of about 5 ~ 9 μm randomly distribute on the lotus leaf surface (Figure 1C; Yong et al., 2017a). Each papilla is further coated with abundant nanorods with a diameter of ~120 nm (Figure 1D). The surface of the lotus leaf is also coated with a layer of wax crystals. The contact area between the lotus leaf surface and water droplets is greatly reduced by the highly rough micro/nanoscale hierarchical structures. An air cushion forms between the water droplet and the rough surface microstructure. The water droplet is at the Cassie wetting state on the lotus leaf surface, as depicted in Figure 1B (Wang and Jiang, 2007; Yong et al., 2017c, 2018c). The repulsive interaction between water and air just allows water to touch the top portion of the surface microstructures. Therefore, the hierarchical microstructures and the waxy crystals endow lotus leaf with superhydrophobicity.

Water droplet on the superhydrophobic lotus leaf has a spherical shape and can easily roll off. Because most dust particles have a stronger ability to adhere to the liquid surface than adhere to a solid surface, the rolling droplets are more likely to pick up the heterogeneous contaminants on the superhydrophobic surface. When the lotus leaf has a slight shake or tilted angle, the spherical water droplets roll down and take the pollutants or dust on the lotus leaf away, leaving a clean path behind the droplets (Figure 1E). As a result, the lotus leaf can be self-cleaned by the water droplets such as raindrops. This phenomenon is called “lotus leaf effect” or “self-cleaning effect” which results from the ultra-low adhesive superhydrophobicity of lotus leaf (Nishimoto and Bhushan, 2013; Yong et al., 2013, 2014b; Ragesh et al., 2014).

Fogging can scatter light and cause poor optical performance for an optical element. Interestingly, mosquitoes possess a fascinating vision even in a humid habitat (Gao et al., 2007). When the fog composed of many tiny water droplets is blown toward the mosquito eyes, it is found that the tiny fog drops are unable to stick on the surface of mosquito eyes (Figure 1F). The mosquito eyes feature superhydrophobic and anti-fogging properties. The mosquito compound eye consists of hundreds of microscale hemispheres (Figure 1G; Gao et al., 2007). These hemispheres are called ommatidia and can act as individual sensory units. The ommatidia with a diameter of 26 μm are uniformly arranged (Figure 1H), with rich fine nanoscale nipples on their surface (Figure 1I). These nipples with a diameter of 101.1 nm are very uniform and organize in a hexagonal non-close-packed array (Figure 1I). It is the combination of the microscale ommatidia and the nanoscale nipples that creates the superhydrophobicity for preventing fog drops (moisture) from adhering to the mosquito eye.

Fly eyes also cannot be wetted in some extremely miry and moist environments (Figure 1J; Sun et al., 2014). The compound eyes (around 5 mm in size) are made up of repeating ommatidia with a diameter of 20 μm (Figures 1K,L; Sun et al., 2014). The surface of ommatidia is covered with abundant bubble-like protuberances with a diameter of ~100 nm (Figure 1M). The fly compound eyes are also superhydrophobic and have the ideal anti-fogging ability.

Fish (inset of Figure 1N) can maintain its skin clean even in oil-contaminated waters. Such underwater oil resistance results from the underwater superoleophobicity of fish scales (Liu et al., 2009; Yong et al., 2018a). Fish scales are mainly composed of protein, calcium phosphate, and a thin layer of mucus. There are many hill-like microstructures orderly arranging on the surface of the fan-shaped fish scales (Figures 1N,O; Yong et al., 2018a). The surface of each convex structure is decorated with abundant nanoscale bleb structures (Figure 1P). The high-surface-energy chemical composition and rough microstructure make fish scales superhydrophilic in the air and become superoleophobic after immersion in water. The oil droplets on the fish scale surface have an oil CA above 150° (inset of Figure 1P). In water, the fish scale is fully wetted by water and a layer of water is trapped between the oil droplet and the fish scales. The trapped water cushion hinders the effective contact between the oil droplet and the fish scales so that the oil is only in contact with the tip of the surface microstructures of fish scales, as shown in Figure 1Q. Such an oil droplet is at the underwater Cassie wetting state on the fish scales (Figure 1Q; Yong et al., 2014a, 2017c, 2018c). As a result, the fish scale has remarkable repellence to oils in water.

The superhydrophobicity and underwater superoleophobicity in nature can provide some inspiration toward endowing MLAs and ACEs with advanced liquid repellence, anti-contamination, and anti-fogging property by the combination of proper surface microstructure and chemical composition (Wang et al., 2015).

## Fabrication of Superwetting MLAs

Rough surface microstructures are required to achieve superwettability (Wang et al., 2015). However, a rough surface structure usually reduces the transparency for an optical material because of the light scatter. To prepare a superwetting MLA, it is important to skillfully integrate a superwetting microstructure on an MLA surface, without weakening the optical performance of the MLA. Two strategies have been proposed to prepare such superwetting MLAs. One is the fabrication of a hybrid pattern which is composed of the MLA domain and the superwetting domain. Another is the direct formation of superwetting nanostructures on the surface of the microlenses.

**Figure 2:**
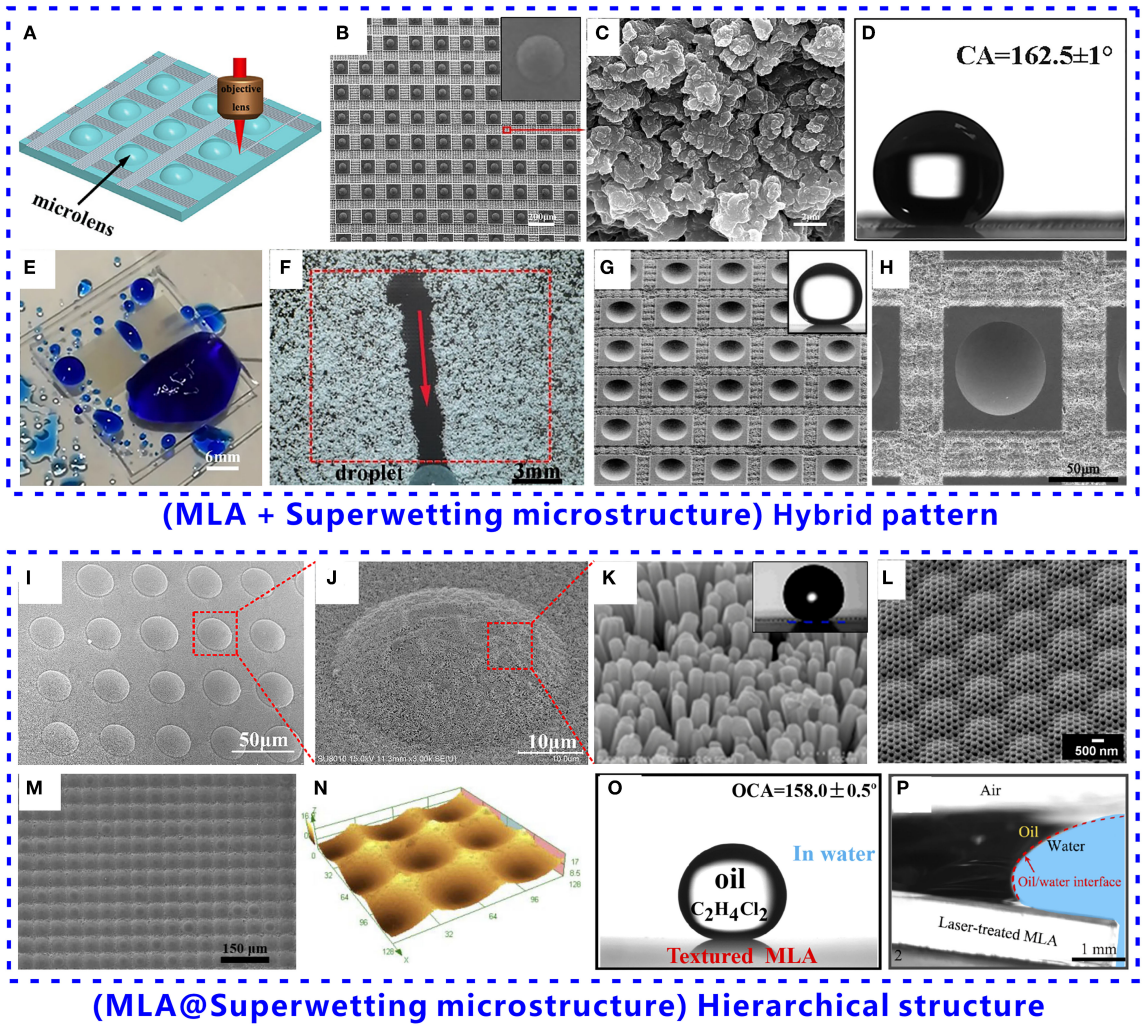
Fabrication of superwetting MLAs and ACEs based on two strategies: **(A–H)** the hybrid pattern composed of the MLA domain and the superwetting domain and **(I–P)** the hierarchical structure of superwetting microstructures on the top surface of the microlenses. Superhydrophobic MLA pattern consisted of a convex MLA and the surrounding superhydrophobic microstructures on a PDMS surface: **(A)** schematic of generating superhydrophobic microstructures between microlenses by selective laser ablation, **(B)** SEM image of the as-prepared MLA pattern, **(C)** SEM image of the laser-induced microstructures on PDMS surface, **(D)** water droplet on the superhydrophobic MLA pattern, **(E)** remarkable water repellence of the superhydrophobic MLA, and **(F)** remarkable self-cleaning function of the superhydrophobic MLA. The inset in **(B)** shows the SEM image of a single microlens of the MLA pattern. **(G,H)** SEM images of an underwater superoleophobic MLA pattern consisted of a concave MLA and the surrounding laser-induced microstructures on a glass substrate. The inset in **(G)** shows an oil droplet on the underwater superoleophobic MLA pattern in a water medium. Superhydrophobic MLA with nanostructure on the top surface of the microlenses produced by crystal growth method: **(I)** SEM image of the hierarchical MLA structure, **(J)** SEM image of the surface of a single microlens, and **(K)** uniform nanorods grown on the surface of microlenses. The inset in **(K)** shows a water droplet on the hierarchical MLA pattern. **(L)** SEM image of the hierarchical MLA fabricated by the sacrificial layer mediated nanoimprinting. Formation of underwater superoleophobic micro/nanostructure on the top surface of the microlenses of an MLA: **(M)** SEM image of the underwater superoleophobic MLA, **(N)** 3D profile of the MLA, **(O)** oil droplet on the MLA surface in water, and **(P)** self-cleaning ability of the underwater superoleophobic MLA as the oil-polluted MLA is dipped into the water. Reproduced from Li M. et al. (2019a) with the permission of the WILEY-VCH. Reproduced from Bian et al. (2020) with the permission of Bian et al. Reproduced from Raut et al. (2015) and Li J. et al. (2019) with the permission of the American Chemical Society. Reproduced from Li M. et al. (2019b) with the permission of Li et al.

Superwetting microstructure can be created on the flat area between microlenses rather than the top surface of the microlenses, making the whole MLA surface finally present superwettability. Li M. et al. (2019a) reported a superhydrophobic MLA pattern consisted of a convex MLA and the surrounding superhydrophobic microstructures on a polydimethylsiloxane (PDMS) substrate. An MLA was firstly obtained by the femtosecond laser wet etching technology and the template replicated method. To achieve superhydrophobicity, rough microstructures were further prepared on the rest flat area between the microlenses by laser direct ablation, as shown in Figure 2A. Figure 2B shows the SEM image of the as-prepared PDMS pattern. The surface of the microlenses is not ablated by laser and thus remains smooth (inset of Figure 2B). The smoothness is important to the imaging capability for a microlens. Each microlens is surrounded by the grid-patterned laser-ablated domain with rough surface microstructures between the microlenses (Figure 2C). The laser-induced PDMS microstructures have great superhydrophobicity. Water droplet on the laser-structured PDMS surface is at the Cassie wetting state (Figure 1B) and can only touch the tip of the surface microstructures. As a result, the hybrid pattern composed of a MLA and the surrounding superhydrophobic microstructure is prepared. A water droplet can maintain a spherical shape with a CA of 161.5° (Figure 2D) on the as-prepared MLA pattern and can roll off easily with a sliding angle (SA) of 0.5°, like on a superhydrophobic lotus leaf. The laser-structured region is enough to provide a repellent effect to water droplets for the hybrid pattern. Therefore, the formation of the surrounding microstructure endows the MLA substrate with superhydrophobicity and ultralow adhesion to water.

The superhydrophobic MLA still has excellent imaging performance because the surface of the microlenses is not treated by laser and remains smooth. As shown in Figure 2E, when a jet of water (blue color) is randomly sprayed onto the superhydrophobic MLA, no water residual leaves on the superhydrophobic MLA, whereas water can stick on the surface of the normal MLA, demonstrating that the as-prepared superhydrophobic MLA has remarkable water repellence (Li M. et al., 2019a). The imaging ability of the superhydrophobic MLA is not affected by the water droplets because water is unable to adhere to the MLA. Similar to a lotus leaf, the superhydrophobicity also endows the MLA with a remarkable self-cleaning function. Once the MLA surface is polluted, the contaminants will be easily removed by water droplets. During the spherical water droplets rolling off on the MLA sample, the droplets concurrently take away the dust and contaminants on their path (Figure 2F; Li M. et al., 2019a).

Similar to the superhydrophobic MLA pattern, Bian et al. (2020) prepared an underwater superoleophobic MLA pattern on a transparent K9 glass substrate through the ingenious combination of the smooth microlenses and the laser-induced rough micro/nanostructures, as shown in Figures 2G,H. The smooth MLA can be used for underwater imaging while the surrounding microstructures endow the sample with underwater anti-oil ability. Underwater oil droplet on the MLA pattern has a CA of 160.0° (inset of Figure 2G) and a SA of 1.5°. The underwater superoleophobicity enables the MLA pattern to have the self-cleaning ability in the water, ensuring its impressive imaging capability even after oil contamination.

Superwetting structures can also be directly created on the top surface of the microlenses, allowing the whole MLA surface to be decorated with a layer of superwetting structures. Li J. et al. (2019) reported a novel manufacturing strategy to prepare superhydrophobic MLA and ACE. A concave MLA was firstly achieved on the elastomer substrate by a template replication. Then, ZnO nanorods were easily grown on the surface of the microlens through the crystal growth method (Figures 2I,J). The uniform nanorods have a length of 202–621 nm and a diameter of 90–127 nm (Figure 2K). The water droplet on the nanostructured MLA surface has a CA of ~161°, so the as-prepared MLA surface exhibits superhydrophobicity (inset of Figure 2K). Such superhydrophobic MLA can be easily extended to a dome-like profile, thus generating a superhydrophobic ACE structure. In addition to the achievement of the superhydrophobicity, the surface reflectance of the MLA is also reduced by the nanostructures, with a decline of ~25% in the wavelength range of 400–800 nm compared with a planar surface. Therefore, the integrated nanostructure endows the MLA and ACE with antireflection and water repellence.

Raut et al. (2015) reported an improved method to fabricate multiscale ommatidia arrays by sacrificial layer mediated nanoimprinting, as shown in Figure 2L. Nanostructures with a size of 200 nm were firstly prepared on a polycarbonate (PC) film by imprinting mold. Then, poly(sodium 4-styrene sulfonate) (PSS) solution was spin-coated on the PC substrate to form a 500 nm thin film. To mold the nanostructured surface into the shape of MLAs, a second imprinting process was implemented on the nanostructured pattern which was encapsulated in the PSS thin film. As a sacrificial layer, the PSS layer protected the underlying nanostructures from buckling or deforming and was finally dissolved away after mold release. As a result, highly uniform multiscale microlenses with nanostructures on their surface were produced (Figure 2L). When the methacrylate-containing resin was used to replicate the multiscale ommatidia arrays on glass, a superhydrophobic MLA was obtained with a water CA of ~151°. The CA hysteresis, which is the difference between advancing CA and receding CA, was as low as 2°, indicating that such superhydrophobic MLA exhibits extremely low adhesion to water droplets (Yong et al., 2017c). The superhydrophobicity endows the as-prepared MLA with an anti-fogging property which is crucial in the retention of the wealthy optical property of the multiscale MLA even in wet and humid conditions.

MLAs also have wide applications in the aqueous environment. The surface pollution by oil contaminations usually weakens the optical imaging ability of the normal MLA. Li M. et al. (2019b) fabricated an underwater superoleophobic MLA with great oil-repellent and self-cleaning abilities in water. An MLA was firstly formed on a commercial K9 glass substrate by a femtosecond laser wet etching method. The surface of the original microlenses is smooth, without any noticeable particles on its surface. To generate micro/nanostructures on the MLA surface, the MLA surface was further ablated by laser. As a result, the whole surface of the laser-treated microlenses is covered with micro-bump structures (about 1 μm in size) (Figure 2M). Even though the microlenses are roughed, they still maintain the lens-curved surface profile (Figure 2N). The formation of microstructures on the microlens surface makes the textured MLA have superhydrophilicity with a water CA of 7.8°. Various oils are repelled by the MLA in water. An oil droplet on the textured MLA has an oil CA of 158.0° (Figure 2O) and an oil SA of 2.0° in a water medium, revealing the underwater superoleophobicity of the structured MLA. The underwater superoleophobicity is attributed to that the oil droplet is at the underwater Cassie state on the textured MLA surface, like an underwater oil droplet on the fish scale (Figure 1Q; Yong et al., 2018c). In a water medium, oil droplets can only touch the peaks of the micro/nanostructures on the MLA surface.

The underwater superoleophobicity endows the MLA with oil resistance and self-cleaning ability. As the oil-polluted MLA is dipped into water, the oil molecules in the surface microstructure are easily replaced by water because of the superhydrophilicity and underwater superoleophobicity of the laser-textured MLA surface (Figure 2P). Under such a process, all the oil contaminants detach from the MLA surface and float onto the water surface, without any oil residues on the MLA surface.

Each approach has its benefits and drawbacks. For the superwetting MLA fabricated by the hybrid pattern method, the surface of its microlenses is not roughed, so the MLA remains original imaging capacity. However, the arrangement of the microlenses is unable to reach a highly close-packed state. Regarding the superwetting MLA based on hierarchical structures, the size of the required nanostructures on the surface of the microlenses should be not too large (usually less than half wavelength of light), otherwise, the nanostructures will cause obvious light scatter.

## Conclusions and Outlook

In conclusion, the recent achievements related to the fabrication of superwetting MLAs and ACEs are reviewed. Inspired by the superwettability in nature, superwetting micro/nanostructures are integrated into the MLA surface, thereby endowing the MLA with anti-liquid and self-cleaning properties. Two strategies are generally utilized to prepare such a superwetting MLA. A superwetting microstructure can be created on the flat area between microlenses, resulting in a hybrid pattern composed of the MLA domain and the superwetting domain. On the other hand, the superwetting nanostructures can also be directly created on the top surface of the microlenses. The resultant MLAs and ACEs exhibit superhydrophobicity or superoleophobicity, which endows those optical components with excellent liquid repellence and self-cleaning function besides good optical performance.

Although several superwetting MLAs and ACEs have been reported, the fabrication of such superwetting optical components is currently still in its infancy. Many development problems need to be solved before practically applying the superwetting MLAs and ACEs in various optical systems. Firstly, poor mechanical/chemical durability of the superwetting micro/nanostructures may result in the decline of the surface superwettability and optical performance for a superwetting MLA after a short period of use. Stable superwettability can extend the service life of the superwetting MLAs and ACEs. Secondly, the influence of the surface micro/nanostructures on surface wettability as well as the optical performance of an MLA should be deeply studied and optimized, which has a positive role in designing and fabricating different superwetting MLAs and ACEs. Finally, how to effectively integrate the superwetting MLAs with other optical components in a micro-optical system is also a technical problem. The adverse effect of the generated superwetting micro/nanostructures on the basic optical function of the micro-optical system should be minimized. We believe that the liquid repellence and self-cleaning function will broaden the applications of the superwetting MLAs and ACEs in the medical endoscope, solar cells, microfluidic system, bioscience research, ocean exploration, and other optical systems that are often used in the humid or liquid environment.

## Author Contributions

FC directed and supervised the research. JY and HB wrote the manuscript. QY and XH contributed toward significant discussions and revised the paper. All authors contributed to the article and approved the submitted version.

## Conflict of Interest

The authors declare that the research was conducted in the absence of any commercial or financial relationships that could be construed as a potential conflict of interest.
